# Red Blood Cell Distribution Width during the First Week Is Associated with Severity and Mortality in Septic Patients

**DOI:** 10.1371/journal.pone.0105436

**Published:** 2014-08-25

**Authors:** Leonardo Lorente, María M. Martín, Pedro Abreu-González, Jordi Solé-Violán, José Ferreres, Lorenzo Labarta, César Díaz, Oswaldo González, Daida García, Alejandro Jiménez, Juan M. Borreguero-León

**Affiliations:** 1 Intensive Care Unit, Hospital Universitario de Canarias, La Laguna, Tenerife, Spain; 2 Intensive Care Unit, Hospital Universitario Nuestra Señora Candelaria, Santa Cruz de Tenerife, Tenerife, Spain; 3 Deparment of Phisiology, Faculty of Medicine, University of the La Laguna, La Laguna, Tenerife, Spain; 4 Intensive Care Unit, Hospital Universitario Dr. Negrín, Las Palmas de Gran Canaria, Gran Canria, Spain; 5 Intensive Care Unit, Hospital Clínico Universitario de Valencia, Valencia, Spain; 6 Intensive Care Unit, Hospital San Jorge, Huesca, Spain; 7 Intensive Care Unit, Hospital Insular, Las Palmas de Gran Canaria, Gran Canaria, Spain; 8 Research Unit, Hospital Universitario de Canarias, La Laguna, Tenerife, Spain; 9 Laboratory Deparment, Hospital Universitario de Canarias, La Laguna, Tenerife, Spain; University of Leicester, United Kingdom

## Abstract

**Objective:**

Higher values of red blood cell distribution width (RDW) have been found in non-surviving than in surviving septic patients. However, it is unknown whether RDW during the first week of sepsis evolution is associated with sepsis severity and early mortality, oxidative stress and inflammation states, and these were the aims of the study.

**Methods:**

We performed a prospective, observational, multicenter study in six Spanish Intensive Care Units with 297 severe septic patients. We measured RDW, serum levels of malondialdehyde (MDA) to assess oxidative stress, and tumour necrosis factor (TNF)-α to assess inflammation at days 1, 4, and 8. The end-point was 30-day mortality.

**Results:**

We found higher RDW in non-surviving (n = 104) than in surviving (n = 193) septic patients at day 1 (p = 0.001), day 4 (p = 0.001), and day 8 (p = 0.002) of ICU admission. Cox regression analyses showed that RDW at day 1 (p<0.001), 4 (p = 0.005) and 8 (p = 0.03) were associated with 30-day mortality. Receiver operating characteristic curves showed that RDW at day 1 (p<0.001), 4 (p<0.001), and 8 (p<0.001) could be used to predict 30-day mortality. RDW showed a positive correlation with serum MDA levels at day 1 and day 4, with serum TNF-α levels at days 4 and 8, and with SOFA score at days 1, 4 and 8.

**Conclusions:**

The major findings of our study were that non-surviving septic patients showed persistently higher RDW during the first week of ICU stay than survivors, that RDW during the first week was associated with sepsis severity and mortality, that RDW during the first week could be used as biomarker of outcome in septic patients, and that there was an association between RDW, serum MDA levels, and serum TNF-α levels during the first week.

## Introduction

Red blood cell distribution width (RDW) is a laboratory index used in the differential diagnosis of anemia. RDW is a simple laboratory test used to evaluate variability in the size and form of red blood cells. Recently, high RDW has been associated with increased mortality in patients with coronary disease [1]–[3], heart failure [4]–[7], pulmonary hypertension [8], acute pulmonary embolism [9], cardiac arrest [10], stroke [11], liver disease [12], [13] and peripheral artery disease [14]. High RDW has been also associated with increased mortality in the general population [15], [16].

RDW has been previously explored in patients with infectious diseases [17]–[22]. Previous studies have reported higher RDW at day 1 in non-surviving than in surviving patients with community acquired pneumonia [19], [20], gram-negative bacteremia [21] and severe sepsis [22], and also at day 3 in the study by Ku et al with gram-negative bacteremia patients [21]. In addition, elevated RDW has been associated with increased mortality in intensive care patients [17], [18], patients with community acquired pneumonia [19], [20], gram-negative bacteremia [21] and severe sepsis [22].

The pathophysiologic mechanisms underlying the association between RDW and mortality are unclear; however, it is possible that its relationship with inflammation and oxidative states plays a role in this [23], [24]. RDW has been linked with inflammation in critically ill patients [23] and with oxidative stress in animal models [24]. In addition, pro-inflammatory cytokines [25]–[27] and oxidative stress [28]–[30] have been associated with increased mortality in septic patients. However, the following questions remain unanswered: 1) Does RDW differ throughout the first week of intensive care between surviving and non-surviving septic patients? 2) Is there an association between RDW during the first week and sepsis severity? 3) Is there an association between RDW during the first week and sepsis mortality? 4) Could RDW during the first week be used as a predictor of outcome in septic patients? 5) Is there an association between RDW and serum malondialdehyde (MDA) as a biomarker of oxidative stress during the first week? And 6) Is there an association between RDW and serum tumor necrosis factor-alpha (TNF-α) levels as a biomarker of inflammation during the first week? The present study sought to answer these questions.

## Methods

### Design and Subjects

We performed a prospective multicenter study of patients with severe sepsis using a post-hoc analysis to examine whether there is an association between RDW and mortality. The study was carried out in six Spanish Intensive Care Units (ICU) after approval by the Institutional Ethic Review Boards of the six hospitals recruiting patients: Hospital Universitario de Canarias (La Laguna, Santa Cruz de Tenerife), Hospital Universitario Nuestra Señora de Candelaria (Santa Cruz de Tenerife), Hospital Universitario Dr. Negrín (Las Palmas de Gran Canaria), Hospital Clínico Universitario de Valencia (Valencia), Hospital San Jorge (Huesca), Hospital Insular (Las Palmas de Gran Canaria). The written informed consent from the patients or from their legal guardians was obtained.

We included intensive care unit (ICU) patients with a diagnosis of severe sepsis according to the International Sepsis Definitions Conference criteria [31]. Exclusion criteria were: age <18 years, pregnancy, lactation, human immunodeficiency virus (HIV), white blood cell count <1,000 cells/µl, solid or hematological tumor, or immunosuppressive, steroid or radiation therapy.

### Variables recorded

The following variables were recorded for each patient: RDW reported as a coefficient of variation (percentage) of red blood cell volume (reference range 10–14.5%), sex, age, diabetes mellitus, chronic renal failure (CRF) defined as glomerular filtration rate less than 60 ml/min per 1.73 m2, chronic obstructive pulmonary disease (COPD), site of infection, creatinine, leukocytes, lactic acid, platelets, international normalized ratio (INR), activated partial thromboplastin time (aPTT), Acute Physiology and Chronic Health Evaluation II (APACHE II) score [32] and Sepsis-related Organ Failure Assessment [SOFA] score [33]. In addition, we measured serum levels of tumor necrosis factor-alpha (TNF-α) to assess inflammation, and serum levels of malondialdehyde (MDA) to assess oxidative stress. We assessed 30-day mortality as the endpoint.

### Blood samples

Blood samples were analyzed on day 1, 4 and 8 of severe sepsis diagnosis for the determination of RDW, and serum MDA and TNF-α levels. Day 1 was considered as the first day that severe sepsis was diagnosed (baseline values). Day 4 was considered as the day after 72 hours had elapsed, and day 8 as the day after 7 days had elapsed since the diagnosis of severe sepsis.

### Determination of serum MDA and TNF- α levels

Serum separator tubes (SST) were used to determine serum MDA and TNF-α levels. Venous blood samples were taken and centrifuged within 30 minutes at 1000 g for 15 min, and the serum was removed and frozen at −80°C until measurement.

The assay of MDA levels was centralized in the Department of Physiology, Faculty of Medicine (University of the La Laguna. Santa Cruz de Tenerife. Spain). Serum MDA levels were measured using thiobarbituric acid-reactive substance (TBARS) method as described by Kikugawa et al [34]. TBARS assay is a general reaction of aldehydes, but the method developed by Kikugawa et al (1992) is selective for MDA for two reasons: 1) It employs a standard curve of pure MDA and the results are expressed only as MDA (with reference to the standard curve of authentic MDA). 2) The selective extraction of MDA in the butanolic phase of the standard curve and in the samples allows the results to be expressed as authentic MDA. In this assay, the TBARS complex is extracted with an organic solvent (n-butanol). This action, over the pink complex, allows high selectivity in the analytical technique. Each sample was placed in a 96-well plate and read at 535 nm in a microplate spectrophotometer reader (Benchmark Plus, Bio-Rad, Hercules, CA, USA). The detection limit of this assay was 0.079 nmol/ml; the intra- and inter-assay CV were 1.82% and 4.01%, respectively.

TNF-α assay were performed in the Laboratory Department of the Hospital Universitario de Canarias (La Laguna, Santa Cruz de Tenerife, Spain) and were measured using a solid-phase, chemiluminiscence immunometric assays kit (Immulite, Siemens Healthcare Diagnostics Products, Llanberis, United Kingdom). The intra- and inter-assay CV were 3.6% and 6.5%, respectively; and detection limit for the assay was 1.7 pg/ml.

### Statistical Methods

Continuous variables are reported as medians and interquartile ranges. Categorical variables are reported as frequencies and percentages. Comparisons of continuous variables between groups were carried out using Wilcoxon-Mann-Whitney test. Comparisons between groups for categorical variables were carried out with chi- square test. The association between continuous variables was carried out using Spearman's rank correlation test. We plotted three receiver operating characteristic (ROC) curves using survival at 30 days as the classification variable and RDW at day 1, 4 and 8 as the prognostic variable. Analysis of survival at 30 days with Kaplan-Meier curve method and comparisons by log-rank test were carried out using RDW percentage lower/higher than 15.5% as the independent variable and survival at 30 days as the dependent variable. Cox regression analyses were applied to determine prediction of 30-day mortality. Hazard ratio and 95% confidence intervals (CI) were calculated as measures of the clinical impact of the predictor variables. A P value of less than 0.05 was considered statistically significant. Statistical analyses were performed with SPSS 17.0 (SPSS Inc., Chicago, IL, USA).

## Results

Comparative baseline values of demographic and clinical parameters between surviving (n = 193) and non-surviving (n = 104) septic patients are shown in Table 1. We found that non-survivors showed higher age and rate of diabetes mellitus compared with survivors.

**Table 1:** Baseline values of patients' demographic and clinical characteristics according survival and non-survival at 30 days.

	Survival (n = 193)	Non-survival (n = 104)	p-value
Gender female – n (%)	60 (31.1)	36 (34.6)	0.60
Age - median years (p 25–75)	57 (45–68)	65 (56–74)	<0.001
Diabetes mellitus – n (%)	52 (26.9)	41 (39.4)	0.04
Chronic renal failure – n (%)	11 (5.7)	12 (11.5)	0.11
Chronic obstructive pulmonary disease – n (%)	32 (16.6)	16 (15.4)	0.87
Ischemic heart disease - n (%)	20 (10.4)	8 (7.7)	0.54
Site of infection			0.75
· Respiratory - n (%)	109 (56.5)	59 (56.7)	
· Abdominal - n (%)	54 (28.0)	29 (27.9)	
· Neurological	5 (2.6)	0	
· Urinary - n (%)	10 (5.2)	6 (5.8)	
· Skin - n (%)	8 (4.1)	4 (3.8)	
· Endocarditis - n (%)	6 (3.1)	5 (4.8)	
· Osteomyelitis - n (%)	1 (0.5)	1 (1.0)	
Microorganism responsible			
· Unknown - n (%)	103 (53.4)	58 (55.8)	0.72
· Gram-positive- n (%)	45 (23.3)	25 (24.0)	0.89
· Gram-negative- n (%)	45 (23.3)	21 (20.2)	0.56
· Fungii- n (%)	4 (2.1)	4 (3.8)	0.46
· Anaerobe- n (%)	1 (0.5)	1 (1.0)	0.99
Bloodstream infection – n (%)	27 (14.0)	13 (12.5)	0.86
Empiric antimicrobial treatment adequate			0.79
· Unknown due to negative cultures- n (%)	103 (53.4)	58 (55.8)	
· Adequate - n (%)	78 (40.4)	37 (35.6)	
· Unknown due to antigenuria diagnosis-n(%)	4 (2.1)	3 (2.9)	
· Inadequate- n (%)	8 (4.1)	6 (5.8)	
Betalactamic more aminoglycoside - n (%) (%)aminoglycoside- n (%)	37 (19.2)	24 (23.1)	0.45
Betalactamic more quinolone - n (%)	101 (52.3)	51 (49.0)	0.63

Comparisons of continuous variables during the first week are showed in table 2. We found that non-survivors showed higher lactic acid serum levels and SOFA score than survivors during the first week.

**Table 2:** Organ dysfunction and laboratory data at day 1, 4 and 8 in 30-day surviving and non-surviving patients. Median (25^th^ -75^th^) percentiles are shown.

Parameters	Survivors	Nonsurvivors	P-value
**Day 1**	**(n = 193)**	**(n = 104)**	
Pa0_2_/FI0_2_ ratio - median (percentile 25–75)	180 (120–270)	168 (96–240)	0.21
Creatinine (mg/dl) - median (percentile 25–75)	1.20 (0.80–1.90)	1.70 (1.00–3.00)	0.003
Bilirubin (mg/dl) - median (percentile 25–75)	0.90 (0.54–1.56)	0.90 (0.50–2.05)	0.71
Leukocytes -median×10^3^/mm^3^ (percentile 25-75)	18.7 (13.9–27.7)	20.2 (15.0–25.7)	0.88
Lactic acid - median mmol/L (percentile 25–75)	1.8 (1.1–3.3)	3.4 (1.6–6.0)	<0.001
Platelets - median×10^3^/mm^3^ (percentile 25–75)	197 (133–272)	136 (76–222)	<0.001
INR - median (percentile 25–75)	1.25 (1.10–1.50)	1.42 (1.14–1.89)	0.005
aPTT - median seconds (percentile 25–75)	32 (28–39)	37 (30–46)	0.001
SOFA score - median (percentile 25–75)	8 (7–11)	11 (9–14)	<0.001
Malondialdehyde - median nmol/ml (percentile 25–75)	2.31 (1.53–3.81)	3.81 (1.97–6.24)	<0.001
TNF-alpha median pg/ml (percentile 25–75)	31.8 (20.1–50.9)	39.0 (18.1–74.8)	0.34
**Day 4**	**(n = 190)**	**(n = 77)**	
Pa0_2_/FI0_2_ ratio - median (percentile 25–75)	236 (194–280)	198 (133–300)	0.01
Creatinine (mg/dl) - median (percentile 25–75)	0.80 (0.70–1.38)	1.45 (0.80–2.02)	<0.001
Bilirubin (mg/dl) - median (percentile 25–75)	0.75 (0.40–1.20)	1.09 (0.40–3.32)	0.17
Leukocytes -median×10^3^/mm^3^ (percentile 25–75)	13.5 (9.8–17.9)	16.2 (10.9–23.5)	0.41
Lactic acid - median mmol/L (percentile 25–75)	0.8 (1.1–1.5)	1.9 (1.5–2.8)	<0.001
Platelets - median×10^3^/mm^3^ (percentile 25–75)	195 (93–272)	98 (54–192)	0.003
INR - median (percentile 25–75)	1.17 (1.06–1.35)	1.34 (1.15–1.59)	<0.001
aPTT - median seconds (percentile 25–75)	31 (27–37)	33 (29–45)	0.03
SOFA score - median (percentile 25–75)	7 (4–10)	10 (7–15)	<0.001
Malondialdehyde - median nmol/ml (percentile 25–75)	2.03 (1.20–3.07)	3.38 (2.08–6.84)	<0.001
TNF-alpha median pg/ml (percentile 25–75)	22.0 (13.7–31.6)	33.5 (26.3–46.1)	<0.001
***Day 8***	**(n = 184)**	**(n = 60)**	
Pa0_2_/FI0_2_ ratio - median (percentile 25–75)	267 (203–370)	200 (150–280)	<0.001
Creatinine (mg/dl) - median (percentile 25–75)	0.70 (0.60–1.00)	1.10 (0.80–2.20)	<0.001
Bilirubin (mg/dl) - median (percentile 25–75)	0.62 (0.40–1.10)	0.50 (0.26–1.67)	0.44
Leukocytes -median×10^3^/mm^3^ (percentile 25–75)	14.0 (10.0–18.9)	14.8 (7.8–24.3)	0.33
Lactic acid - median mmol/L (percentile 25–75)	1.0 (0.8–1.2)	1.1 (0.9–1.9)	<0.001
Platelets - median×10^3^/mm^3^ (percentile 25–75)	234 (113–358)	119 (64–203)	<0.001
INR - median (percentile 25–75)	1.10 (1.00–1.25)	1.16 (1.03–1.37)	0.005
aPTT - median seconds (percentile 25–75)	28 (26–32)	37 (28–45)	<0.001
SOFA score - median (percentile 25–75)	4 (2–7)	10 (6–13)	<0.001
Malondialdehyde - median nmol/ml (percentile 25–75)	1.74 (1.30–3.28)	2.57 (1.50–3.70)	<0.001
TNF-alpha median pg/ml (percentile 25–75)	16.9 (12.0–29.2)	26.7 (17.4–50.3)	0.03

PaO_2_/FIO_2_ = pressure of arterial oxygen/fraction of inspired oxygen; INR = International normalized ratio; aPTT = Activated partial thromboplastin time; SOFA = Sepsis-related Organ Failure Assessment score; TNF = tumor necrosis factor (TNF)

**Figure 1:**
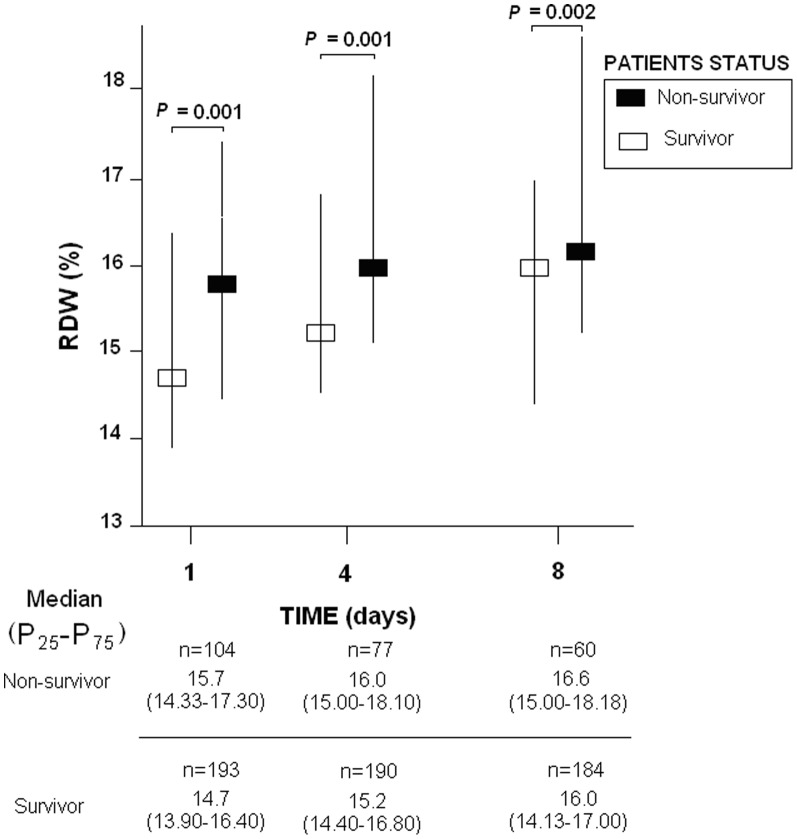
Red blood cell distribution width (RDW) in survivor and non-survivor septic patients.

We found higher RDW in non-surviving than in surviving septic patients at day 1 (p = 0.001), day 4 (p = 0.001) and day 8 (p = 0.002) of ICU admission (Figure 1).

Table 3 shows correlations between RDW, serum MDA and TNF-α levels and SOFA score at days 1, 4 and 8. We found a positive correlation between RDW and serum MDA levels at day 1 (p<0.001) and day 4 (p = 0.009); between RDW and serum TNF-α levels at day 4 (p = 0.002) and day 8 (p = 0.007); and between RDW and SOFA score at day 1 (p = 0.007), day 4 (p = 0.002) and day 8 (p<0.001). In addition, we found a positive association between RDW and age (rho = 0.13; p = 0.02).

**Table 3:** Correlations between red blood cell distribution width (RDW), malondialdehyde, tumor necrosis factor (TNF)-α, lactic acid and Sepsis-related Organ Failure Assessment (SOFA) score at day 1, 4 and 8.

	Day 1	Day 4	Day 8
Malondialdehyde	rho = 0.21; p<0.001	rho = 0.18; p = 0.009	rho = 0.13; p = 0.06
TNF-alpha	rho = 0.01; p = 0.91	rho = 0.21; p = 0.002	rho = 0.27; p<0.001
SOFA score	rho = 0.21; p = 0.007	rho = 0.21; p = 0.002	rho = 0.31; p<0.001
Lactic acid	rho = 0.09; p = 0.13	rho = 0.23; p = 0.001	rho = 0.27; p<0.001

Patients with a medical history of CRF showed higher RDW (p<0.001); however, there were no significant differences in RDW according to gender, history of diabetes mellitus, COPD or ischemic heart disease (Table 4).

**Table 4:** Red blood cell distribution width (%) on day 1 according the gender and personal history of some diseases.

	Non	Yes	p-value
Gender female	15.0 (14.0–16.4)	15.3 (14.0–17.2)	0.38
Diabetes mellitus	15.1 (14.0–16.5)	15.1 (14.0–17.1)	0.46
Chronic renal failure	15.0 (14.0–16.4)	17.0 (15.4–19.4)	<0.001
Chronic obstructive pulmonary disease	15.1 (14.0–16.7)	14.9 (14.0–16.5)	0.94
Ischemic heart disease	15.1 (14.0–16.7)	14.9 (14.0–16.4)	0.90

Cox regression analyses showed that RDW at day 1 (p = 0.001), 4 (p = 0.01) and 8 (p = 0.04) were associated with 30-day mortality, controlling for age, gender, and history of diabetes mellitus, CRF, COPD and ischemic heart disease (Table 5).

**Table 5:** Cox regression analysis models to predict 30-day mortality at day 1, 4 and 8 of sepsis evolution.

	Odds Ratio	95% Confidence interval	P-value
**Model 1:**			
RDW at day 1	1.13	1.05–1.22	0.001
Age	1.03	1.01–1.04	<0.001
Gender female	1.10	0.72–1.66	0.66
Diabetes mellitus	0.81	0.53–1.23	0.32
Chronic renal failure	0.87	0.46–1.65	0.67
Chronic obstructive pulmonary disease	1.37	0.79–2.39	0.27
Ischemic heart disease	1.51	0.72–3.16	0.27
**Model 2:**			
RDW at day 4	1.11	1.03–1.21	0.01
Age	1.02	1.01–1.04	0.02
Gender female	1.08	0.66–1.76	0.76
Diabetes mellitus	0.63	0.39–1.03	0.06
Chronic renal failure	0.83	0.40–1.72	0.62
Chronic obstructive pulmonary disease	1.23	0.64–2.35	0.53
Ischemic heart disease	1.36	0.61–3.05	0.45
**Model 3:**			
RDW at day 8	1.10	1.01-1.21	0.04
Age	1.02	1.01–1.04	0.04
Gender female	0.92	0.53–1.62	0.78
Diabetes mellitus	0.57	0.33–1.01	0.052
Chronic renal failure	0.84	0.36–1.94	0.69
Chronic obstructive pulmonary disease	1.19	0.58–2.47	0.64
Ischemic heart disease	1.36	0.57–2.33	0.49

RDW = red blood cell distribution width.

**Figure 2:**
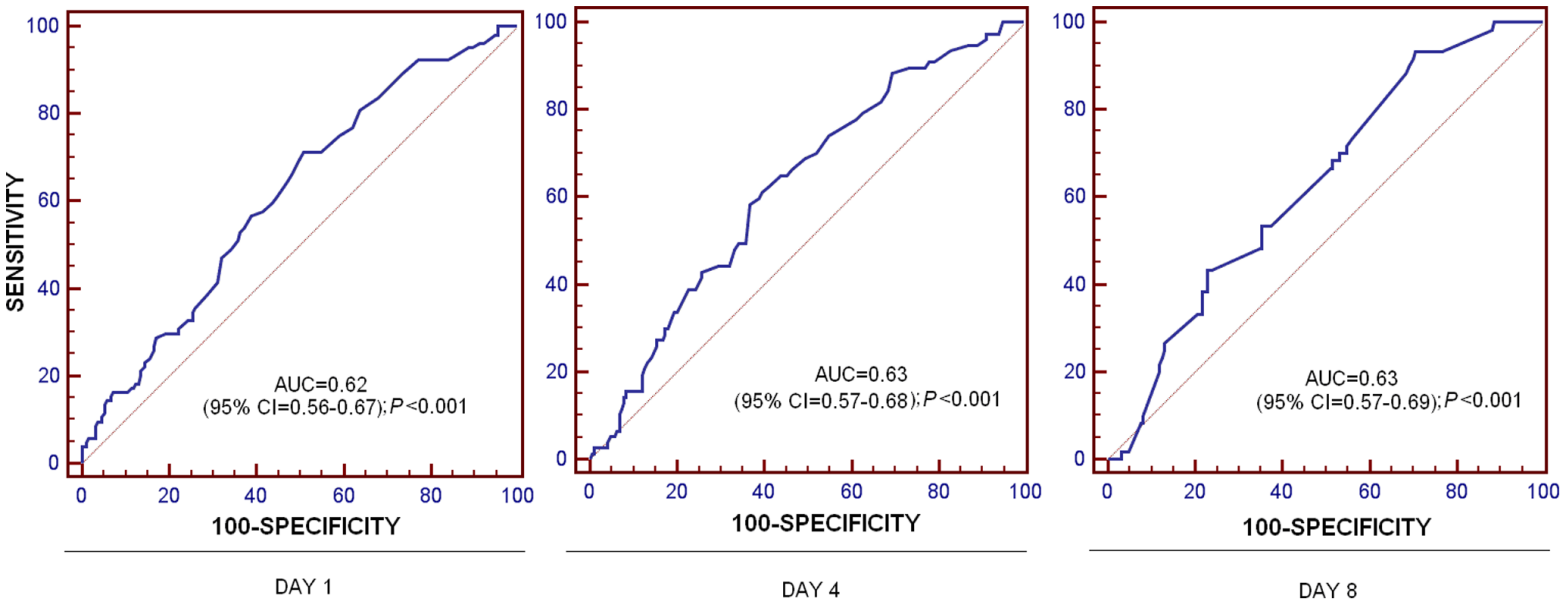
Receiver operation characteristic (ROC) curve using red blood cell distribution width (RDW) at day 1, 4 and 8 as predictor of mortality at 30 days in septic patients.

ROC analyses showed that RDW at day 1 (p<0.001), 4 (p<0.001), and 8 (p<0.001) could be used to predict outcomes in septic patients (Figure 2).

**Figure 3:**
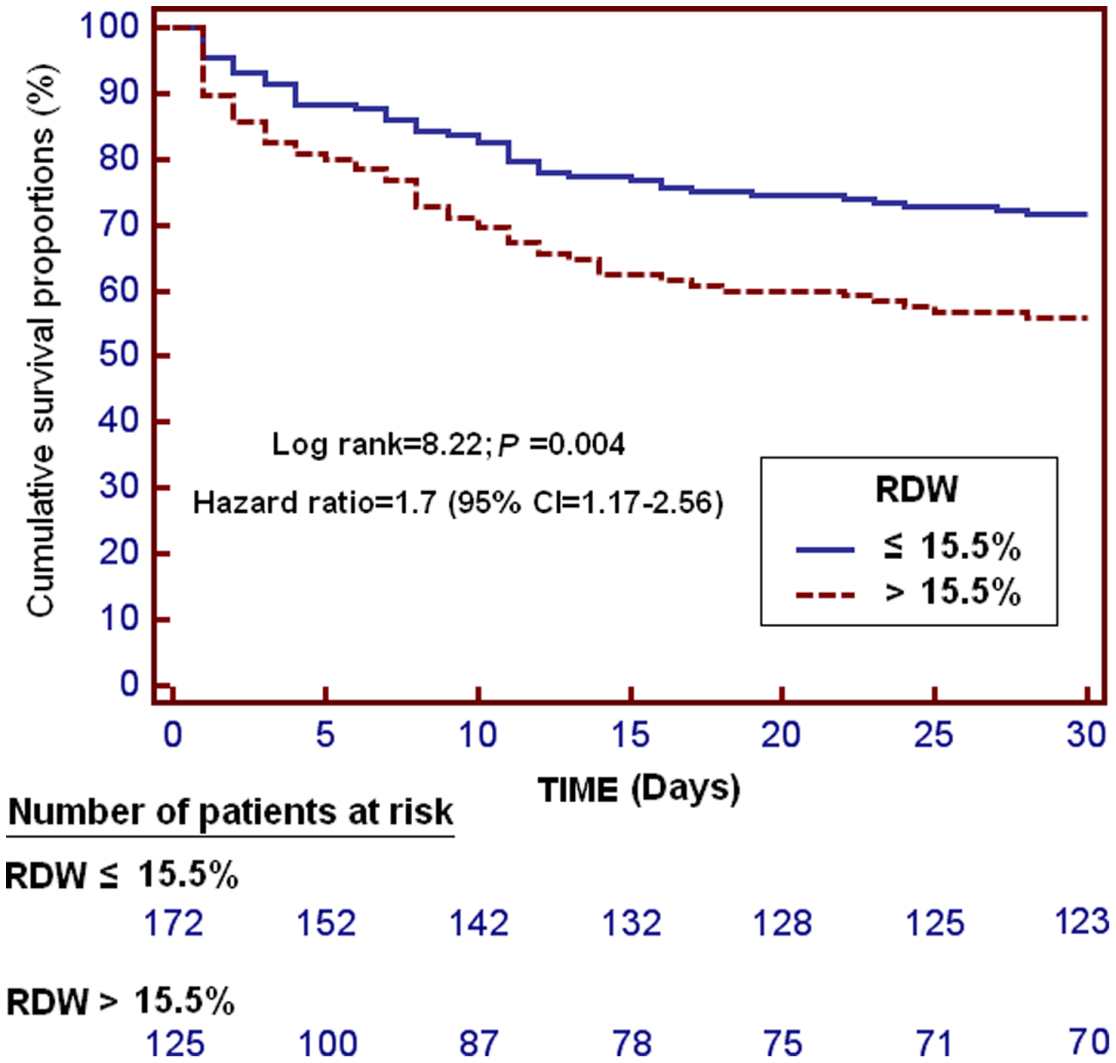
Survival curves at 30 days using red blood cell distribution width (RDW) higher or lower than 15.5%.

Kaplan-Meier survival analysis showed that patients with RDW higher than 15.5% had a lower probability of survival at day 30 (log-rank = 8.22; Hazard ratio = 1.7 (95% CI = 1.17–2.56); p = 0.004) than patients with a lower percentage (Figure 3).

## Discussion

The major novel findings of our study were that non-surviving septic patients showed persistently higher RDW during the first week of ICU stay than survivors, that RDW during the first week was associated with sepsis severity and mortality, that RDW during the first week could be used to predict outcome in septic patients, and that there was an association between RDW, serum MDA and serum TNF-α levels during the first week.

Non-surviving septic patients had significantly higher RDW than survivors at days 1, 4 and 8. Previous studies have reported higher RDW at day 1 in non-survivors than in survivors with community acquired pneumonia [19], [20], gram- negative bacteremia [21] and severe sepsis [22], and also at day 3 in a study by Ku et al with gram-negative bacteremia patients [21]. In addition, we report for the first time that non-surviving septic patients also showed significantly higher RDW than survivors at day 8.

RDW on day 1 has been associated with early mortality in septic patients [19]–[22]. We also found this association; what the present study adds is that RDW at day 4 and 8 is also associated with early mortality in these patients.

Some authors have found an association between RDW and proinflammatory cytokines [23], [35], and between RDW and other markers of inflammation such as erythrocyte sedimentation rate (ESR) or C-reactive protein (CRP) [36]. In addition, an association between circulating TNF-α levels and increased mortality has been found in septic patients [25]. We here report for the first time an association between RDW and serum TNF-α levels in septic patients during the first week.

In animal models, RDW has been linked with the presence of certain molecules related to oxidative stress such as reactive oxygen species (ROS), superoxide dismutase (SOD) and glutathione peroxidase [24]. In addition, an association between oxidative state and mortality has been found in septic patients [28]–[30]. We here report for the first time an association between RDW and serum MDA levels during the first week of ICU stay in septic patients.

Interestingly, we observed an association between RDW and SOFA score at days 1, 4 and 8. To our knowledge, this link between RDW and sepsis severity throughout the first week of ICU stay has not been previously reported.

In addition, RDW was associated with age and history of CRF, but not with gender, history of diabetes mellitus, COPD or ischemic heart disease. These associations were previously found in patients with stable coronary artery disease [37]. Osadnik et al found that RDW was associated with mortality after controlling for all these comorbities [37]. We found that RDW at days 1, 4 and 8 of sepsis evolution was associated with early mortality after controlling for age, gender, and history of diabetes mellitus, CRF, COPD and ischemic heart disease. Thus, like Osadnik et al, we believe that the association between RDW and comorbidities does not entirely explain the increase in early mortality shown by patients with higher RDW [37]. Currently, it remains unclear why RDW is associated with mortality; however, it is possible that the association of RDW with inflammation and oxidative states plays a role.

Another interesting finding was that RDW at days 1, 4 and 8 of sepsis evolution could be used as a biomarker of prognosis in severe septic patients according to the results of ROC analyses. In addition, Kaplan-Meier analysis showed that those patients with RDW higher than 15.5% had a 70% higher risk of death in the first 30 days than those with a lower RDW.

The strengths of our study were the large sample size; and that was reported information about RDW, and inflammation and oxidative stress throughout the first week of ICU stay. However; the present study has certain limitations. First, we did not report reticulocyte count and blood smear data. Second, RDW could be influenced by iron, folate, and vitamin B12 and these variables were not included in the present analysis. Third, we reported data on serum of TNF-α and MDA and it could be interesting to investigate other cytokines and molecules related to oxidative stress.

## Conclusions

The most relevant and new findings of our study were that non-surviving septic patients showed persistently higher RDW during the first week of ICU stay than survivors, that RDW during the first week was associated with sepsis severity and mortality, that RDW during the first week could be used as biomarker of outcome in septic patients, and that there was an association between RDW, serum MDA levels and serum TNF-α levels during the first week. Thus, since RDW determination is inexpensive, it could be used routinely as a biomarker of early mortality in septic patients.
